# Automated Detection of Positioning Errors in Bitewing Radiographs Using Deep Learning

**DOI:** 10.1002/cre2.70504

**Published:** 2026-11-04

**Authors:** Katharina Alves Rabelo, Zimo Huang, Eduardo Delamare, Shwetha Hegde, Jinman Kim, Vesna Miletic

**Affiliations:** ^1^ Dentomaxillofacial Radiology, Sydney Dental School, Faculty of Medicine and Health The University of Sydney Camperdown New South Wales Australia; ^2^ School of Computer Science, Faculty of Engineering The University of Sydney Camperdown New South Wales Australia; ^3^ Restorative and Reconstructive Dentistry, Sydney Dental School, Faculty of Medicine and Health The University of Sydney Camperdown New South Wales Australia

**Keywords:** convolutional neural network, deep learning, dental digital radiography, imaging errors

## Abstract

**Objectives:**

Bitewing radiographs (BWs) are essential diagnostic tools in dentistry, but are often affected by positioning errors, compromising their diagnostic value and increasing retake rates. This study aimed to develop and evaluate a deep learning‐based automated system for detecting and classifying BW type (premolar/molar), side (right/left), and common positioning errors in a controlled mannequin‐based setting.

**Methods:**

A total of 403 BWs were collected from dental student assessments acquired from dental radiology mannequins. BWs were consensus‐labeled by dentomaxillofacial radiology experts for type, side, and the most common positioning errors. A balanced data set supported training and validation. Convolutional neural network architectures, including ResNet and DenseNet variants, were trained from scratch and evaluated as independent classification tasks using stratified fivefold cross‐validation. Performance was assessed using accuracy, precision, recall, F1 score, and area under the curve, reported as mean ± standard deviation across folds. A confusion matrix was generated for multi‐class cone‐cutting error (CCE) severity classification.

**Results:**

The system achieved accuracies of 95.8% for BW type classification, 99.0% for side classification, 96.3% for CCE detection, and 93.4% for interproximal overlap detection. Multi‐class CCE classification achieved an accuracy of 79.3%, with reliable identification of “critical” and “minimal” errors. Detection of incorrect receptor placement achieved an accuracy of 73.2%.

**Conclusions:**

The CNN‐based system showed strong performance for bitewing type, side, binary cone‐cutting error, and interproximal overlap classification in mannequin‐acquired radiographs. Performance was more limited for multi‐class cone‐cutting severity and incorrect receptor placement. These findings support the potential use of deep learning for radiographic quality assessment in education and quality‐control workflows. A validation study using patient‐acquired bitewing radiographs from clinical settings is required before clinical deployment.

## 1 Introduction

The bitewing radiograph (BW) is a common radiographic technique in dentistry due to its ability to show crowns of opposing posterior teeth and the alveolar crest in one image. For optimal BW acquisition, the receptor can be oriented horizontally or vertically and must be positioned parallel to the buccal and lingual tooth surfaces. Additionally, the X‐ray beam must be directed through the interproximal spaces and perpendicular to the receptor (Mallya and Lam 2018). The geometry of the horizontal and vertical angulations, combined with appropriate receptor placement, provides a clear view of the interproximal surfaces without overlapping structures (Mallya and Lam 2018; Whaites and Drage 2013). Despite the availability of positioning devices for the BW technique, the receptor position and the accurate beam alignment with the receptor and anatomical landmarks largely rely on the operator's technical skills. Therefore, bitewing radiographs are susceptible to a range of positioning errors, also referred to as technical errors, that may compromise diagnostic interpretation. These errors can increase the frequency of retakes, leading to additional radiation exposure, higher costs, and delays in dental procedures.

Despite advancements in Dentomaxillofacial Radiology (DMFR), including the development of digital receptors, enhanced X‐ray units, and positioning devices, positioning errors continue to be reported in the literature as one of the most prevalent causes of unacceptable intraoral radiograph quality. A recent literature review reveals that retake rates for intraoral, extraoral, and cone‐beam computed tomography (CBCT) imaging range from 5% to 20% due to imaging errors. In particular, BWs had an average rejection rate of 11.25%, with positioning errors and patient movement frequently identified as the main causes (Yeung and Wong 2021). In another study, improper angulation (26.1%) and incorrect receptor placement (11.2%) have been identified as the most prevalent positioning errors attributed to intraoral radiograph retakes (Acharya et al. 2015). Furthermore, in periapical radiographs, overlapping of proximal surfaces (Dastgir Bhatti et al. 2020; Peker and Alkurt 2009) has been reported as one of the most frequent imaging errors, further emphasizing the impact of suboptimal receptor positioning and X‐ray beam angulation inconsistencies on intraoral radiographs.

A large number of recent studies have demonstrated that AI models can significantly enhance radiographic interpretation. In particular, deep learning (DL) models using Convolutional Neural Networks (CNNs) have shown high performance when applied to computer vision tasks such as detection, classification, and segmentation (Hung et al. 2022; Chaves et al. 2024; Gonzalez et al. 2024; Yuce et al. 2023). A systematic review found DL models trained on BW data sets achieved accuracies ranging from 71.11% to 94.5% for the detection of interproximal caries (Mahizha et al. 2025).

The accuracy variability in caries detection models could be attributed to differences in data set selection. Some studies excluded radiographs with positioning errors, such as improper angulation or image distortion (Chaves et al. 2024; García‐Cañas et al. 2023), as well as those with cone‐cutting errors, horizontal overlap, receptor placement errors, motion, or artifacts that could hinder diagnosis (Gonzalez et al. 2024; Karakuş et al. 2024). Additionally, other studies explicitly stated that only high‐quality images were included (Estai et al. 2022), while others did not mention any form of image quality assessment as part of the inclusion or exclusion criteria (Bayrakdar et al. 2022). The natural, biological variability in human subjects adds complexity and may reduce the accuracy of the models. Standardizing anatomical structures and exposure settings, such as in BWs taken from mannequins, contributes to explaining the relationship between the accuracy of the AI model in detecting a pathology and specific parameters, such as positioning errors.

A recent study has shown that a CNN (dentalXrai Pro, dentalXrai Ltd.) has improved the diagnostic accuracy of clinicians in detecting enamel carious lesions (Arsiwala‐Scheppach et al. 2024). While these models often achieve high performance metrics, their results are largely based on radiographs free from imaging errors. However, in daily dental practice, positioning errors are common, posing a significant challenge to the robustness and generalizability of DL models trained on strictly curated data sets. Recent studies highlight the fact that the performance of DL models is dependent on the complexity of the characteristics of the dental imagery data set used for training and validation stages (Krois et al. 2021). One study explicitly showed outputs from the CNN model Inception‐ResNet‐v2, in which areas of interproximal overlap, a common positioning error, were misclassified as caries (Estai et al. 2022). Radiographs with variable levels of positioning errors may introduce similar complexity and negatively affect models trained under ideal conditions.

Based on these findings, the introduction of a DL‐based tool for the detection of positioning errors has the potential to enhance AI research on BWs by improving the management of data set heterogeneity. Clinically, this tool could assist dental practitioners with image diagnostic quality before interpretation and support correction of positioning errors, improving radiographic acquisition. This study intends to demonstrate that a DL‐based tool can explicitly detect and classify positioning errors in BW radiographs. It uniquely addresses an important, but largely unexamined, source of data set heterogeneity in AI research. The study separates image acquisition quality from diagnostic content. In so doing, the proposed approach provides a methodological input for developing more robust and generalizable AI models for BW analysis. Therefore, the aim of the study was to assess the performance of an automated system of DL models developed for classifying BW type (premolar/molar), side (right/left), and detecting common positioning errors in BWs acquired from a dental radiology mannequin.

## 2 Methods

### 2.1 Study Design

This retrospective study was reviewed and approved by the University Human Ethics Committee (2024/HE000846). The study applies CNN algorithms to automatically classify bitewings according to the type (premolar and molar), side (right and left), and to detect and categorize common positioning errors.

### 2.2 Data Set

BW radiographs were acquired from an adult dental radiology mannequin during regular assessment periods in the discipline of DMFR at the University, in 2021 and 2022. These radiographs were obtained by students enrolled in the Bachelor of Oral Health and Doctor of Dental Medicine programs. The BWs were taken using a Heliodent Plus intraoral X‐ray device (Dentsply Sirona, Bensheim, Germany) with a focal spot size of 0.4 mm and a pre‐programmed BW exposure setting (tube voltage: 70 kV, tube current: 7 mA, exposure time: 0.10 s), using intraoral Photostimulable Phosphor plate (PSP) receptors (Air Techniques, Melville, New York, USA) and cardboard loop film holders (ADM, n.d.).

A total of 403 BWs from 3 different mannequins of the same X‐ray Phantom (Nissin, Minami‐ku, Kyoto, Japan) were collected by different students in different assessment periods. The selected BWs were uploaded to the Research Data Store networked drive in. jpg format and 1980×1486 resolution.

A senior investigator and lecturer in DMFR with over 8 years of experience selected the radiographs and classified the images based on type (premolar or molar), side (right or left), and the presence of specific positioning errors. Two other investigators— also lecturers in DMFR from SDS—then assessed the selected images. A consensus method was used to classify and label the BWs according to the following positioning errors: cone‐cutting error (CCE); interproximal overlap and incorrect receptor placement.

All images were analyzed until investigators reached a consensus on the presence of each positioning error. This was then considered the ground truth for training and validating the AI models.

#### 2.2.1 Type and Side of Bitewing

The type of BW was determined based on the region of interest (ROI) visible in the image. In this study, it was determined that to be classified as a premolar bitewing (PMBW), the image must show the entire mesial half of the first maxillary premolar. For a molar bitewing (MBW), at least half of the most posterior erupted third molar, or the entire distal surface of the last erupted molar, must be visible. Examples of MBW and PMBW are shown in Figure 1. BW images were further classified as right or left based on the orientation of the image and the anatomical structures visible.

**Figure 1:**
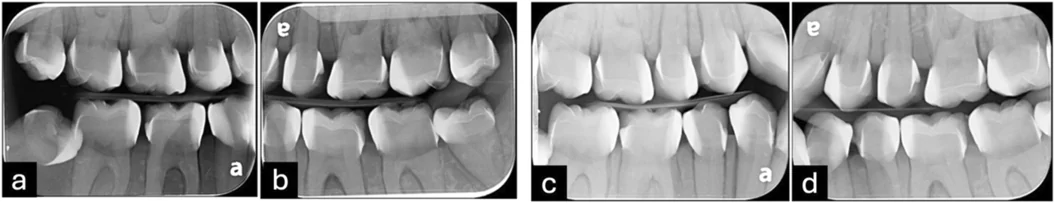
Images representing the types and sides of BWs. (a) Right molar, (b) left molar, (c) right premolar, and (d) left premolar.

#### 2.2.2 Cone Cutting Error

Cone‐cutting error (CCE) in BW radiographs occurs when part of the image appears as a well‐defined, radiopaque, non‐exposed area due to improper alignment of the X‐ray beam with the receptor. A binary classification was performed, categorizing BWs into two groups: presence and absence of CCE. Subsequently, a multi‐class classification was performed on BWs with the presence of CCE, categorized into three classes based on its extent: (Minimal) CCE that does not affect the ROI; (Severe) CCE partially affecting the ROI, compromising three or fewer interproximal areas; and (Critical) CCE significantly affecting the ROI (resulting in a non‐diagnostic radiograph), where more than three interproximal areas are not visible. Examples of BWs demonstrating the three classes of CCE are shown in Figure 2. All BWs classified with a critical CCE (non‐diagnostic) did not undergo further assessment for other positioning errors included in this study.

**Figure 2:**
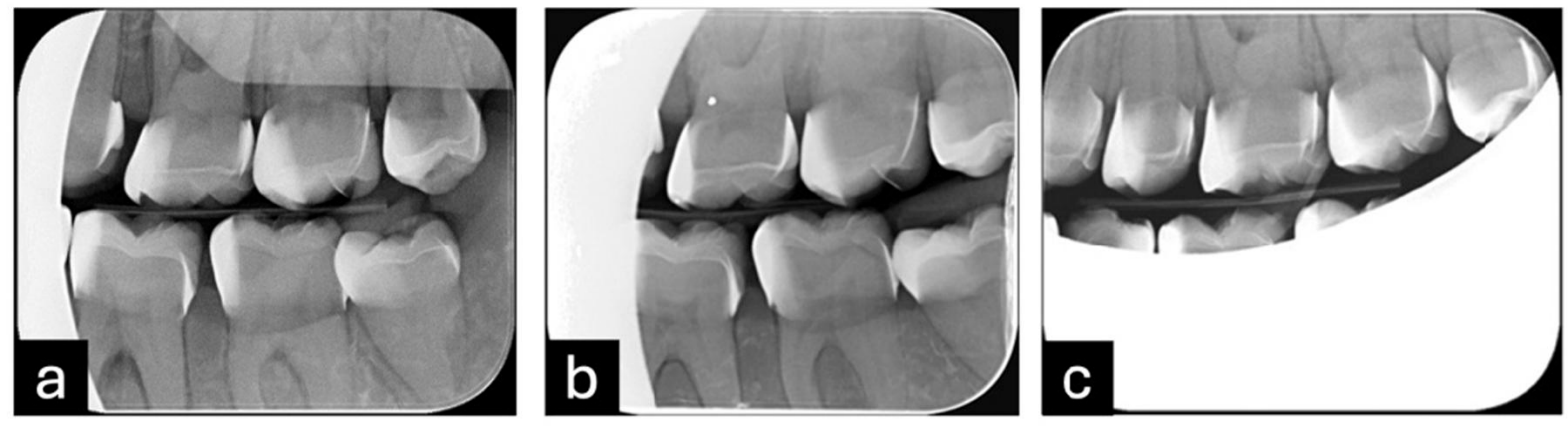
Images representing the types of CCEs. (a) Minimal, (b) severe, and (c) critical.

#### 2.2.3 Interproximal Overlap

Interproximal overlap in BWs occurs when the proximal surfaces of adjacent teeth appear superimposed. BWs were categorized based on the presence or absence of interproximal overlap. For this assessment, interproximal overlaps were defined as those extending beyond the contact point. In PMBWs, interproximal overlap between the mandibular canine and first premolar was not considered a positioning error. Similarly, in MBWs, interproximal overlap around partly erupted third molars was not considered an error as seen on Figure 3.

**Figure 3:**
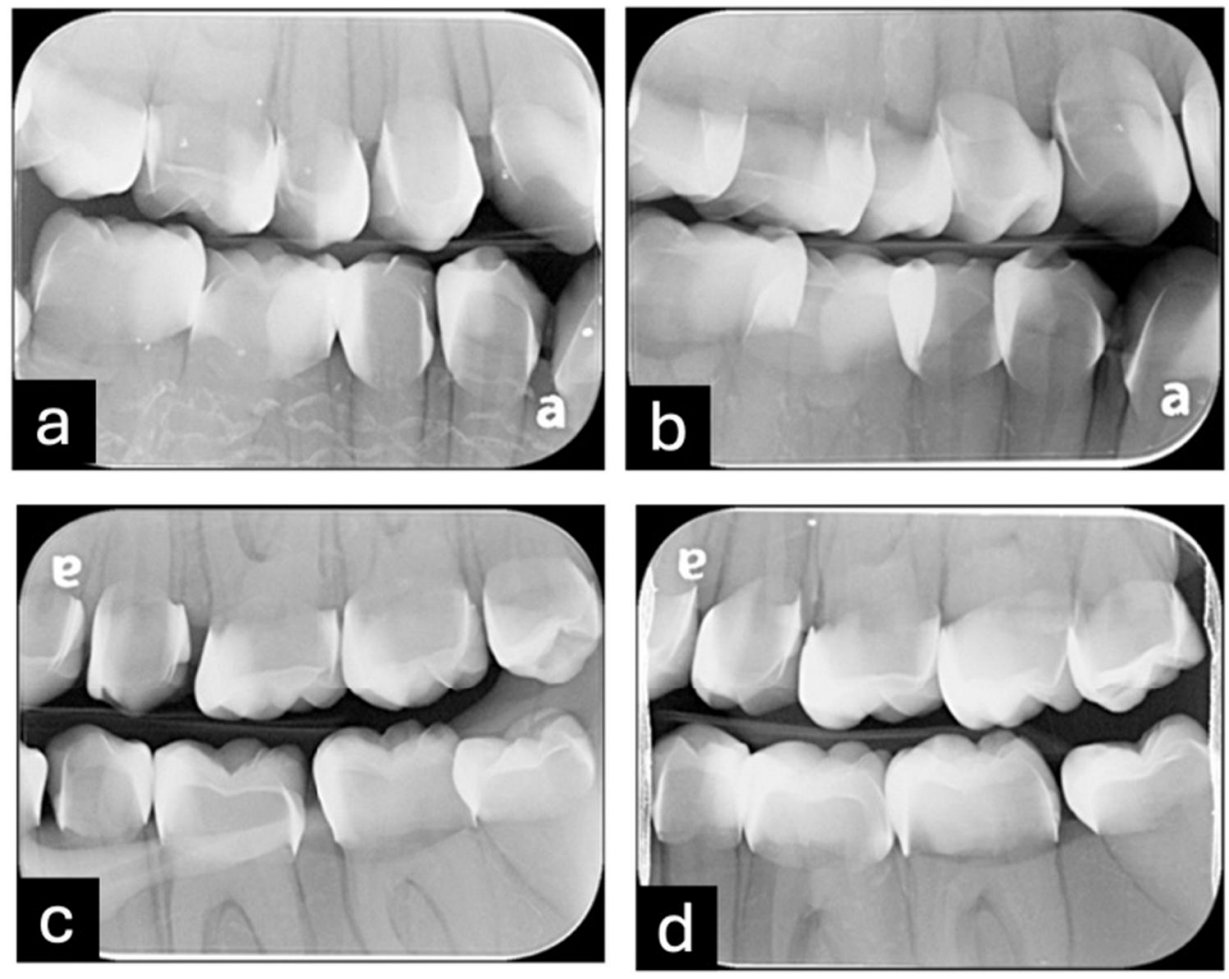
Images representing BWs with interproximal overlap positioning error. (a and b) Right premolar BWs with interproximal overlaps, and (c and d) left molar BWs with interproximal overlaps.

#### 2.2.4 Incorrect Receptor Placement

In BWs, the receptor should be placed so that the upper and lower teeth are evenly captured in the image. Accordingly, the position of the occlusal plane can be used to evaluate receptor placement. A template was used for this assessment (Figure 4). The receptor placement was considered correct when the occlusal plane extended into the green band area of the template (Figure 5a,c). If the occlusal plane extended into the yellow band area of the template, the receptor placement was considered incorrect (Figure 5b,d). If the occlusal plane extended outside the yellow band area of the template, the film placement was considered unacceptable.

**Figure 4:**
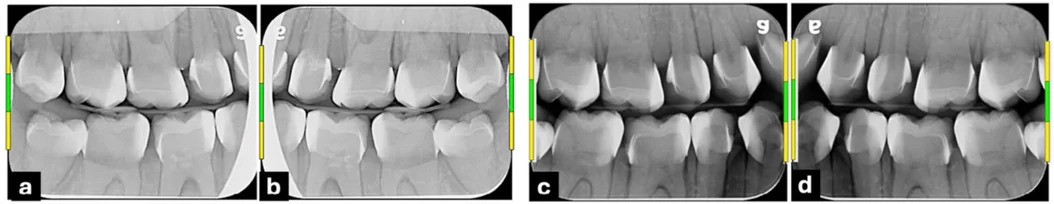
Templates used for assessment of incorrect receptor placement. (a) Right molar, (b) left molar, (c) right premolar, and (d) left premolar.

**Figure 5:**
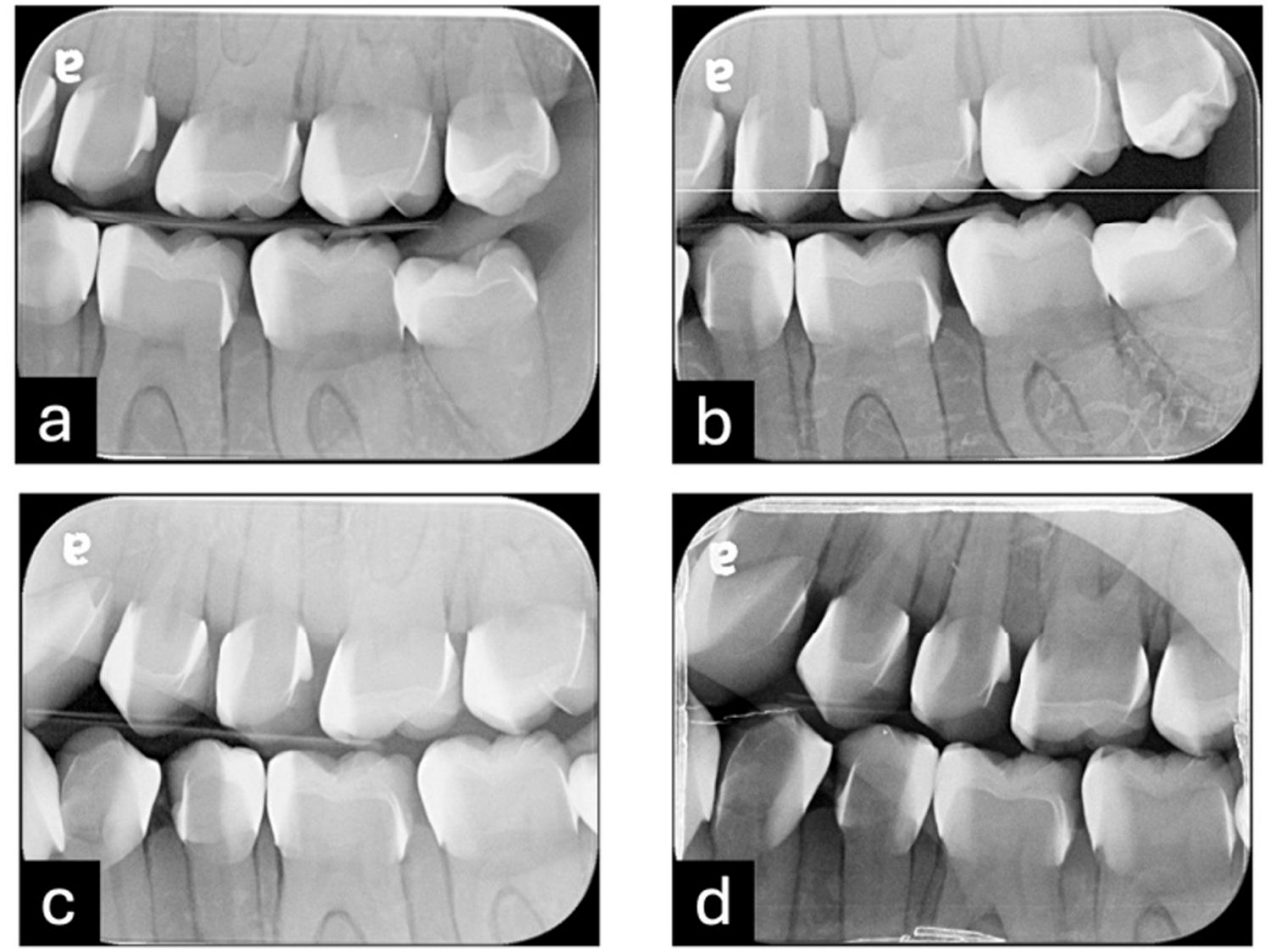
BWs representing the receptor placement classification. (a and c) Correct receptor placement, and (b and d) incorrect receptor placement.

### 2.3 Experimental Setup

For the automated detection of the positioning errors, a structured pipeline of sequential steps was implemented to enhance the AI model's efficiency. This pipeline included six tasks designed to classify BW radiographs based on five criteria: type, side, CCE, interproximal overlap, and incorrect receptor placement. The CCE was divided into two sub‐tasks using both binary and multi‐class classification methods.

The resultant data set of 403 BWs was used to classify images according to type (premolar or molar) and side (right or left). The presence and absence of CCE were evaluated across the entire data set (403 BWs). Additionally, a multi‐class classification was conducted on BWs exhibiting CCE (179 BWs), with the cases grouped into three classes according to their extent (minimal, severe, and critical CCE). Furthermore, based on the CCE multi‐class classification, all critical CCE (non‐diagnostic) (70 BWs) images were excluded from the other two positioning error assessments, interproximal overlap and incorrect receptor placement. For the incorrect receptor placement task, one additional BW was excluded as it was considered non‐diagnostic due to a partial crown cut‐off caused by improper receptor placement. The BW class distribution for each task is shown in Table 1.

**Table 1:** Summary of evaluated tasks, class distributions, and best‐performing models with hyperparameters.

Task	Class distribution			Total	Model	Hyperparameters	
						Learning rate	Batch size
BW type	Premolar (208)		Molar (195)	403	DenseNet169	5e−05	8
BW Side	Right (173)		Left (230)	403	DenseNet121	5e−04	8
CCE (binary)	Present (179)		Absent (224)	403	DenseNet161	1e−04	4
CCE (multi‐class)	Critical (70)	Severe (61)	Minimal (48)	179	DenseNet121	5e−04	4
Interproximal overlap	Present (171)		Absent (162)	333	DenseNet161	5e−05	4
Incorrect receptor placement	Present (155)		Absent (177)	332	ResNet34	5e−04	4

All BWs were pre‐processed by resizing the JPEG images to 512 × 512 pixels. Pixel values were scaled to a consistent range across all images, and mean‐variance normalization was applied to ensure uniform input for the CNNs. The classification models were trained from scratch for 200 epochs using an NVIDIA GeForce RTX 2080 Ti. No data augmentation was applied. Each task was treated independently and evaluated using stratified fivefold cross‐validation to ensure that class distribution remained consistent across folds. Cross‐validation was performed at the image level, with each radiograph assigned to only one fold; therefore, no identical image appeared in both training and validation sets.

A set of CNNs, including ResNet34, ResNet50, DenseNet121, DenseNet161, and DenseNet169, was used as the baseline models for all classification tasks. Models were trained using the Adam optimizer, with Binary Cross‐Entropy loss for binary tasks and Cross‐Entropy loss for the three‐class task, using default PyTorch weight initialization (Paszke et al. 2019), and without learning rate scheduling or additional regularization. For each task, the same predefined training configurations, including learning rate and batch size, were applied across all CNN models to allow fair comparison. The best‐performing model and its selected hyperparameter configuration for each task are summarized in Table 1. Random seeds were not fixed. The implementation used standard and stable PyTorch and scikit‐learn APIs available in 2024 and did not rely on deprecated or experimental version‐specific features. Therefore, the pipeline is expected to be robust to minor version variations.

### 2.4 Performance Metrics

The CNNs’ predictions were evaluated against the labeled ground truth using multiple performance metrics. These metrics included **accuracy** (the proportion of correctly predicted instances across all classes), **recall** (the proportion of actual positive cases correctly identified, also known as sensitivity), **precision** (the proportion of true positive predictions among all predicted positives), and the **F1 score** (the harmonic mean of precision and recall, providing a balanced measure of model performance). For each metric, results are presented as the mean ± standard deviation across the five folds. Additionally, the **area under the curve (AUC)** was calculated to evaluate the model's ability to distinguish between classes. To further evaluate classification performance, a **confusion matrix** was generated for the classification model applied to the multi‐class classification of CCE.

## 3 Results

The performance of the CNN classifier DenseNet169, which distinguishes between MBWs and PMBWs (the BW type classification task), and the performance of the DenseNet121 model, which categorizes BWs as right or left (the BW side classification task), is summarized in Table 2.

**Table 2:** Performance of BW type (molar vs. premolar) and side (left vs. right) classification models (mean ± standard deviation).

Class distribution	BW type			BW side		
	Premolar	Molar	Total	Right	Left	Total
Number of BWs	208	195	403	173	230	403

**Metrics**			**Average**			**Average**
Precision (%)	95.7 ± 3.5	95.9 ± 3.7	95.8 ± 3.0	98.8 ± 1.5	99.1 ± 1.2	99.0 ± 0.6
Recall (Sensitivity) (%)	96.2 ± 3.6	95.4 ± 3.8	95.8 ± 3.0	98.8 ± 1.6	99.1 ± 1.2	99.0 ± 0.6
F1 Score (%)	95.9 ± 2.9	95.6 ± 3.1	95.8 ± 3.0	98.8 ± 0.7	99.1 ± 0.5	99.0 ± 0.6
AUC (%)	99.3 ± 1.0	99.3 ± 1.0	99.3 ± 1.0	99.3 ± 0.7	99.3 ± 0.6	99.3 ± 0.7
Accuracy (%)			95.8 ± 3.0			99.0 ± 0.6

*Note:* Model used: DenseNet169 Model for type classification and DenseNet121 Model for Bitewing Side Classification.

The CCE was evaluated through two classification tasks: binary and multi‐class. The binary classification task aimed to distinguish between the absence and presence of CCE, while the multi‐class classification task further categorized the extent of CCE in the ROI into three classes: Minimal, Severe, and Critical. The CCE binary classification task using DenseNet161 showed excellent performance, achieving an overall accuracy of 96.3%. For the multi‐class classification, the DenseNet121 model achieved an overall accuracy of 79.3%, with the highest performance observed in the “critical” class. The evaluation metrics for the binary and multi‐class classifications are shown in Table 3. The confusion matrix shown in Figure 6 illustrates the performance of the DenseNet‐121 in classifying the extent of CCE in three classes. The matrix highlights the model's higher accuracy in identifying the “Critical” and “Minimal” classes, while showing decreased performance for the “Severe” class. Examples of CCE misclassifications are shown in Figure S1.

**Table 3:** Performance of binary and multi‐class classification models for CCE (mean ± standard deviation).

Class distribution	Binary classification (DenseNet161)			Multi‐class classification (DenseNet121)			
	Present	Absent	Total	Critical	Severe	Minimal	Total
Number of BWs	179	224	403	70	61	48	179

**Metrics**			**Average**				**Average**
Precision (%)	98.8 ± 1.6	94.5 ± 2.3	96.6 ± 1.6	87.3 ± 2.0	71.4 ± 11.8	76.9 ± 6.2	78.6 ± 5.5
Recall (sensitivity) (%)	92.7 ± 3.2	99.1 ± 1.2	95.9 ± 1.9	88.6 ± 10.8	65.6 ± 4.2	83.3 ± 11.6	79.2 ± 5.8
F1 Score (%)	95.7 ± 2.1	96.7 ± 1.5	96.2 ± 1.8	87.9 ± 5.1	68.4 ± 6.2	80.0 ± 8.2	78.8 ± 5.5
AUC (%)	98.6 ± 2.0	98.6 ± 2.0	98.6 ± 2.0	94.9 ± 1.9	72.2 ± 3.8	94.5 ± 4.6	87.2 ± 2.6
Accuracy (%)			96.3 ± 1.8				79.3 ± 5.7

**Figure 6:**
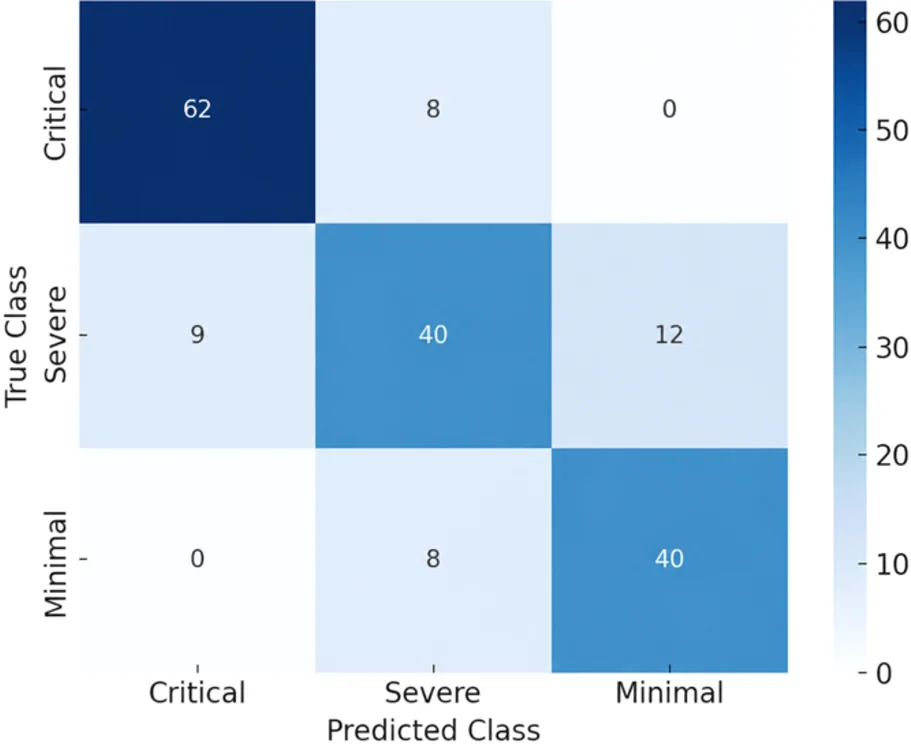
Confusion matrix representing the predicted versus actual classification outcomes for the DenseNet‐121 classifier applied to BWs to classify the extent of CCE in three classes. (Minimal) CCE that does not affect the region of interest (ROI); (Severe) CCE partially affecting the ROI, compromising three or fewer interproximal areas; and (Critical) CCE significantly affecting the ROI (resulting in a non‐diagnostic radiograph), where more than three interproximal areas are not visible.

The CNN classifier demonstrated strong performance in detecting interproximal overlap, achieving an overall accuracy of 93.4%. The model maintained consistent performance across key metrics, with average precision, recall, and F1 score all reaching 93.4%. The AUC was 97.8%, indicating a high ability to distinguish between images with and without interproximal overlap. These results are shown in Table 4.

**Table 4:** Performance of interproximal overlap and incorrect receptor placement classification models (mean ± standard deviation).

Class distribution	Interproximal overlap			Incorrect receptor placement		
	Present	Absent	Total	Present	Absent	Total
Number of BWs	171	162	333	155	177	332

**Metrics**			**Average**			**Average**
Precision (%)	93.6 ± 6.3	93.2 ± 4.2	93.4 ± 5.2	72.6 ± 10.2	73.7 ± 7.4	73.1 ± 8.3
Recall (sensitivity) (%)	93.6 ± 3.8	93.2 ± 6.9	93.4 ± 5.3	68.4 ± 10.1	77.4 ± 11.3	72.9 ± 8.2
F1 Score (%)	93.6 ± 5.0	93.2 ± 5.5	93.4 ± 5.2	70.4 ± 8.7	75.5 ± 8.4	72.9 ± 8.3
AUC (%)	97.8 ± 2.3	97.8 ± 2.3	97.8 ± 2.3	79.4 ± 8.0	79.4 ± 8.0	79.4 ± 8.0
Accuracy (%)			93.4 ± 5.2			73.2 ± 8.3

*Note:* Model Used: Interproximal overlap: DenseNet161; Incorrect Receptor Placement: ResNet34.

The performance of the ResNet34 model for the receptor placement binary classification task is summarized in Table 4. The model achieved an overall accuracy of 73.2%. The F1 score reflects a balance between precision and recall and is slightly higher for the “Present” class (75.5) than for the “Absent” class (70.4). This indicates that the model was more effective at correctly identifying cases without incorrect receptor placement than at detecting cases in which incorrect receptor placement was present. The AUC was 79.4%, indicating moderate overall performance.

Examples of false‐positive and false‐negative classifications for interproximal overlap and incorrect receptor placement are shown in Figure S2.

## 4 Discussion

The CNN classification models performed well in binary classification tasks for BW type (premolar and molar), side (right and left), and the positioning errors: CCE (presence and absence) and interproximal overlap (presence and absence), achieving accuracies of 95.8%, 99%, 96.3%, and 93.4%, respectively.

Recent studies on quality enhancements of intraoral radiographs using AI algorithms have primarily focused on post‐processing techniques (Moran et al. 2022; Latke and Narawade 2024). However, while such models enhance image clarity, they do not address positioning errors, which remain a significant cause of non‐diagnostic radiographs. This study focuses on positioning errors such as CCE, interproximal overlap, and improper receptor placement, as they are among the most prevalent causes of unacceptable image quality in BW radiographs, as evidenced by previous research (Yeung and Wong 2021; Acharya et al. 2015).

A recent systematic review found that the complexity of a radiograph is one of the factors influencing errors in dental radiograph interpretation, which depends on the type (BW, periapical, CBCT) and the quality of the radiograph (image contrast, presence of imaging errors) (Hegde et al. 2024). A previous study classified BWs based on diagnostic quality by categorizing them as diagnostic or non‐diagnostic, based on the presence of interproximal overlaps (Barayan et al. 2022). However, no studies to date have explored the applicability of AI tools for the detection of specific imaging errors for either clinical or research purposes. The present work fills this gap and demonstrates the good performance of CNN classification models in BW analysis. Our findings support the hypothesis that AI‐based detection tools can effectively identify imaging errors. As suggested by a recent review, such tools have the potential to address documented sources of data set heterogeneity in dental imagery, therefore enhancing the robustness, generalizability, and interpretability of future AI downstream tasks (Delamare et al. 2024).

The selection of mannequin‐based radiographs taken by students as the data set for this study offers a standardized setting that ensures consistent anatomical structures while maintaining variability in execution due to differences in student techniques. This controlled approach is particularly advantageous for assessing technical errors, as it eliminates patient‐related variability, allowing for a more focused evaluation of acquisition‐related faults.

The present results demonstrate, as a key advantage, the potential of these classifiers to be integrated into dental imaging management software, reducing the time spent correctly mounting images and detecting common positioning errors. An automated classification tool could prevent radiograph mounting errors during image processing, reducing misinterpretations and treatment errors. Integrating the type and side of BW tasks into a CDSS in dentistry helps define ROI. This allows region‐specific imaging error detection, improving the accuracy of error identification and analysis. By recognizing positioning errors upfront, the CDSS can determine a radiograph's diagnostic usefulness before attempting to detect any oral pathology. Lastly, beyond clinical applications, these classification tasks can also be integrated into interactive dental learning resources to help students recognize and correct mounting and positioning errors.

Previous studies have explored similar approaches for side classification in BW radiographs. A previous study reported that a ResNet‐34 model achieved 97.56% accuracy in recognizing inverted images (upside‐down) using a relatively small data set (Chen et al. 2020). A recent study employed DenseNet‐121, pre‐trained on the ImageNet data set with transfer learning and fine‐tuning, achieving 100% accuracy in distinguishing left and right in a 1000 BWs data set (Ayhan et al. 2024). These studies consistently demonstrate good results. In contrast, our study employed DenseNet‐121 without transfer learning or fine‐tuning, using a smaller data set of 403 BWs and considering multiple positioning errors. Despite this raw approach, the model demonstrated excellent classification performance, with an overall accuracy of 99.0%. These results should be viewed in light of the BWs taken from mannequins, which may have contributed to this high overall accuracy.

While the consensus in the literature indicates the use of transfer learning and fine‐tuning for improved model performance with small data sets (Dalkıran et al. 2023; Iman et al. 2023), the findings in our study challenge this assumption, showing that a model trained from scratch without relying on a pretrained model can still achieve high accuracy in right‐left classification in a small data set of mannequin's BW radiographs. In that respect, our study has used mannequin radiographs; further investigation in clinical data should be conducted to confirm such findings.

Although the use of a positioning device with a locator ring can minimize the prevalence of CCEs, it remains one of the most frequently reported causes of diagnostically unacceptable images, leading to re‐exposures (Yeung and Wong 2021). In this study, a multi‐class classification was intentionally used to directly compare with the binary classification performance metrics. CCEs can compromise the ROI to various degrees, for example, if the CCE affects the area of interest in a premolar/molar BW, the image is non‐diagnostic. Therefore, we propose an AI‐driven workflow that first identifies and classifies the CCE as a first step, excludes all images with critical CCE as the second step, then evaluates other positioning errors, and lastly detects oral pathologies.

The best classification model employed for the multi‐class classification for CCE, DenseNet121, showed lower accuracy than the best model for binary classification, DenseNet161, where the accuracy decreased from 96.2% to 79.3%. It is expected, as we increase the class number and keep the same data set size the accuracy tends to decrease (Luo et al. 2019). The analysis of images with the wrong predictions suggests that these results may be attributed to the absence of a pre‐defined ROI. A potential strategy to improve model performance in CCE multi‐class classification is the introduction of premolar and molar subclasses within each CCE type. This approach could be implemented using segmentation or object detection models beforehand to determine the ROI, or alternatively, through manual annotation with these subclasses.

The AI model's confusion matrix analysis reveals its strengths and areas for improvement in classifying CCE by the extent to which they affect the region of interest (ROI). Overall, the model is most accurate at recognizing the extremes, “Minimal” and “Critical” CCE, while it sometimes confuses “Severe” CCE with the other two categories. The observation of the radiographs with incorrect predictions suggests that the primary issue lies in the ROI not being defined beforehand, prior to assessing CCE severity across the three classes. This pattern underscores the need for further refinement, such as adjusting decision thresholds, which means changing the cut‐off values the model uses when deciding which class a case belongs to, to more accurately distinguish “Severe” CCE from its minimal or critical counterparts. This demonstrates, as suggested by a previous study (Büttner et al. 2024), that the level of reporting for different positioning errors may need to be considered at the object level or pixel level, therefore employing other types of computer vision tasks, such as segmentation and object detection tasks.

One of the most critical positioning errors in BWs is the interproximal overlap, which can compromise the assessment of proximal tooth surfaces, particularly in the diagnosis of caries. This error may arise from incorrect horizontal angulation or variations in tooth positioning. Because interproximal overlap can undermine diagnostic performance, studies have excluded BWs with this error when training/testing AI models (Chaves et al. 2024; Gonzalez et al. 2024; García‐Cañas et al. 2023; Van Nistelrooij et al. 2024; Panyarak et al. 2023; Bayati et al. 2025; Magat et al. 2024; Köse and Hoşgör 2025). Unfortunately, the performance of these models cannot be reliable in clinical routine, as ideal radiographs without this positioning error are rare. The classifier model, DenseNet161, trained in this study, achieved a 93.4% accuracy in detecting BWs to detect the absence or presence of interproximal overlap. This represents a significant step forward in creating models capable of detecting overlap in a specific interproximal space to alert the pathology detection AI tools to this positioning error, improving the diagnostic accuracy and clinical practice routine.

Incorrect receptor placement was labeled using a classification template based on the vertical orientation of the long axis of the image receptor in relation to the floor. The correct receptor placement in BWs, the occlusal plane appears flat or rising upward from the midline, reflecting the natural curve of Spee. Any variation from this expected pattern was used to identify and classify incorrect receptor placement. Despite a modest overall accuracy of 73.2%, the model demonstrated a moderate level of performance, as reflected in the F1 score, which balances both precision and recall. The F1 score was slightly higher for the “present” class (75.5%), indicating a stronger ability of the model to correctly identify actual incorrect receptor placement. The findings indicate that while the model is more effective at correctly identifying cases without incorrect receptor placement than at detecting cases in which incorrect receptor placement was present, suggesting room for further refinement to improve sensitivity.

The data set used was relatively small; however, it was carefully selected and structured to ensure class balance, which is essential for fair model evaluation. A potential strategy to increase data set size while preserving class balance is the use of synthetic bitewing images for model pre‐training, followed by fine‐tuning on real data, as demonstrated in a previous study (Tolstaya et al. 2025).

This study focused on classification models, which may not be optimal for detecting all types of positioning errors; however, they are commonly used as the initial stage before further optimization. If satisfactory performance is achieved through whole‐image analysis, classification represents a preferable strategy. It minimizes errors introduced by additional preprocessing and requires fewer computational resources than segmentation or object detection CNN tasks (Zhao et al. 2024). Additionally, the study was conducted using radiographs obtained from mannequins, which, although standardized, do not fully capture the variability seen in clinical settings.

A limitation of this study is the need to downsample high‐resolution radiographic images to 512 × 512 pixels, which introduces some loss of detail. Direct processing of full‐resolution images with standard CNN architectures is often constrained by GPU memory limitations, and uniform input image sizes are required to ensure stable and reliable training. However, this approach introduces extra processing steps, increasing the real‐time clinical deployment and the risk of information loss. In our experiments, most clinically relevant structures (e.g., tooth structures and the alveolar bone) remain discernible at reduced resolution, and we suggest this is evident with our strong model performance. In addition, prior studies have shown that models trained on downsampled images often achieve comparable performance to those trained on full‐resolution images for classification and segmentation tasks (Haque et al. 2023).

The integration of these models into various settings would support experts in clinics, research, and education by identifying positioning errors. These models could recommend a retake, flag reduced diagnostic confidence, assist student feedback, or filter images before training. These models could be incorporated into AI diagnostic multi‐agent systems. The sequential approach of these systems, simulating the step‐by‐step reasoning of a human expert, enables a series of specialized agents to perform a more reliable analysis. A recent study demonstrated a significant improvement in performance, achieving near‐perfect detection of pathologies in chest X‐rays using an agentic AI system (Chen et al. 2025). False negatives should be identified for critical cone‐cutting errors or severe overlap, as this would lead to non‐diagnostic images passing unnoticed, while false positives could lead to unnecessary retakes and additional radiation exposure.

Future research should expand on the proposed workflow for automatic identification of positioning errors by incorporating other model architectures or alternative CNN approaches. This study provides a foundation for large clinical trials employing multi‐center data sets with patient‐acquired bitewing radiographs to reflect clinical variability, aiming to enhance the robustness and generalizability of the results.

## 5 Conclusions

Our results indicate that an automated system of DL models in a controlled mannequin‐based setting is capable of satisfactorily detecting and classifying most of the common positioning errors in BW. For BW type (premolar and molar), side (right and left), and the positioning errors: CCE (presence and absence) and interproximal overlap (presence and absence), achieving accuracies in the range of 93%–99%. Regarding the incorrect receptor placement error, the model demonstrated a moderate level of performance, with an overall accuracy of 73.2%.

## Author Contributions

**Katharina Alves Rabelo:** conception and design of the study, data acquisition, analysis, and interpretation of data, drafting the original manuscript, and revising the manuscript. **Zimo Huang:** feedback on the concept and methodology, method implementation, analysis and interpretation of data, review and editing of the manuscript. **Eduardo Delamare:** conception and design of the study, data acquisition, analysis and interpretation of data, review and editing of the manuscript. **Shwetha Hegde:** data acquisition, analysis and interpretation of data, review and editing of the manuscript. **Jinman Kim:** feedback on the concept and methodology, analysis and interpretation of data, critical revision and editing of the manuscript. **Vesna Miletic:** feedback on the concept and study design, analysis and interpretation of data, critical revision and editing of the manuscript.

## Funding

The authors have nothing to report.

## Ethics Statement

This study was approved by the Ethics Committee of the University of Sydney (approval number: 2024/HE000846). Ethical approvals were granted in accordance with the ethical standards outlined in the Declaration of Helsinki.

## Conflicts of Interest

The authors declare no conflicts of interest.

## Supporting information


Supporting File 1

Supporting File 2

## Data Availability

Data that support the findings of this study are available on request from the corresponding author.
